# Utility of FEV_1_/FEV_6_ index in patients with multimorbidity hospitalized for decompensation of chronic diseases

**DOI:** 10.1371/journal.pone.0220491

**Published:** 2019-08-02

**Authors:** Shakeel Komal, Lluis Simon, Gemma Grau, Aina Mateu, Maria de la Asunción Villaverde, Alex de la Sierra, Pere Almagro

**Affiliations:** 1 Multimorbidity Unit, Internal Medicine Service, University Hospital Mutua de Terrassa, University of Barcelona, Terrassa, Spain; 2 Internal Medicine Department, University Hospital Mutua de Terrassa, University of Barcelona, Terrassa, Spain; National Yang-Ming University, TAIWAN

## Abstract

Spirometry remains essential for the diagnosis of airway obstruction. Nevertheless, its performance in elderly hospitalized patients with multimorbidity can be difficult. The aim of this study is to assess the utility of the COPD-6 portable device in this population. We included all patients hospitalized for exacerbation of chronic diseases in a medical ward specialized in the care of multimorbidity patients, between September 2017 and May 2018. A questionnaire including sociodemographic, cognitive and functional impairment, among other variables, was completed the last day of admission. Subsequently, patients attempted to perform three valid respiratory manoeuvres with the COPD-6 device and then conventional spirometry. A total of 184 patients were included (mean age of 79.61 years, 55% men). Forty-seven (25.54%) patients were able to perform complete spirometric manoeuvres and 99 (53.8%) could perform a valid FEV1/FEV6 determination. The inability to perform a valid spirometry was related with the patient’s age, functional physical disability, cognitive impairment or the presence of delirium or dysphagia during admission. Only 9% of patients with a Mini Mental Cognitive Examination (MMEC) lower than 24 points could perform a valid spirometry. Of the patients with an MMEC < 24 points and unable to perform spirometry, 34% were able to complete the FEV1/FEV6 manoeuvres. No differences were found in the Charlson index, multimorbidity scale, number of domiciliary drugs, or length of stay between those patients able and those not able to perform respiratory manoeuvres. The agreement between the values for FEV1 measured with COPD-6 and those observed in the spirometry was good (r: 0.71; p<0.0001). Inability to perform a valid spirometry during hospitalization in elderly patients with multimorbidity is frequent and related with functional and cognitive impairment. FEV1/FEV6 determination using the COPD-6 portable device allows an important percentage of the patients with limitations to complete spirometric measurement.

## Introduction

Improvement in socioeconomic status, alongside progress in the management of chronic diseases and better care for decompensation, is associated with a sustained increase in life expectancy in developed and emerging countries [1]. This longer survival is linked to a marked increase in the prevalence of elderly patients with multiple concomitant chronic illnesses. Recent studies have shown that the mean of these chronic diseases in the general population over 80 years is about 5 pathologies, rising to 8 in hospitalized patients [2,3].

This fact has led to the search for new terminologies and concepts to replace the classic definition of comorbidity–understood as a primary disease with other secondary pathologies associated with it–given the difficulty in deciding which one is the predominant disease in an individual patient. Regarding these new proposed concepts, several authors have suggested the term multimorbidity as being more appropriate, defined as the presence of two or more concomitant long-term diseases in the same patient [4].

Chronic respiratory diseases account for an important percentage of multimorbidity, although compared with other chronic diseases pulmonary disorders are often underdiagnosed [2–8]. The causes of this misdiagnosis are, among others, the lack of suspicion and the need to perform a valid spirometry to confirm the diagnosis, in many of them, of Chronic Obstructive Pulmonary Disease (COPD). Although spirometry is a simple and widely available technique, its performance in frail elderly patients can be difficult, especially due to the inability to maintain expiratory flow until complete exhalation. Depending on the population studied, between 20 and 80% of elderly patients are unable to complete a satisfactory spirometry [9,10].

In recent years, several handheld spirometers have been developed. Usually these spirometers measure the FEV_1_/FEV_6_ ratio, requiring less effort and allowing the recovery of the 25% of patients unable to complete forced vital expiration [11,12]. These devices have proven useful in the screening for respiratory obstruction and have shown an excellent correlation with conventional spirometry [13]. However, current evidence of the utility of FEV_1_/FEV_6_ for the diagnosis of airway obstruction in multimorbid patients hospitalized for decompensation of chronic diseases is scarce [12].

Our main objective was to study the utility and reliability of FEV_1_/FEV_6_ in the diagnosis of airway obstruction in patients hospitalized with multimorbidity, and the variables associated with the inability to successfully perform valid manoeuvers to determine FEV_1_/FEV6 with COPD-6 and FEV_1_/FVC with conventional spirometry.

## Methods

The present study was performed between September 1, 2017 and May 31, 2018, in a hospitalization medical ward specialized in the care of multimorbidity patients in the University Hospital Mutua de Terrassa, in Terrassa, Spain. We included the first admission of all patients hospitalized for exacerbation of chronic disease with two or more criteria of multimorbidity according to the functional definition of the Andalusia Health Department. This classification includes 15 chronic pathologies selected for relevant severity or impact on daily living activities (Table 1) [14]. On the last day of hospitalization patients or their caregivers completed a questionnaire specifically designed for the study. The questionnaire was administered by trained investigators. A previous pilot study was performed to guarantee their viability [12]. This questionnaire included medical and socioeconomic variables, smoking history, prior diagnosis of chronical respiratory disease, results of the most recent spirometry if available, and domiciliary treatment. Multimorbidity assessment was evaluated using the multimorbidity classification of the Andalusia Health Department, the Charlson index, a scale for other comorbid conditions not included in the Charlson index, and the PROFUND index [14–17]. The PROFUND index is a multicomponent prognostic scale designed and validated for patients with multimorbidity, and which includes variables such as age, presence of caregiver, dyspnoea, delirium during admission, physical functional dependence, and number of hospitalizations in the previous year. The score ranges from 0 to 36 points [17] “S1 Table”. Physical functional status was assessed with the Barthel index, while cognitive status was measured using the Pfeiffer test and the Mini Mental Cognitive Examination in the Spanish version of Lobo et al. (MMCE) [18–20]. MMCE is analogous to the original Mini Mental State Examination of Folstein et al. and maintains the same structure of evaluation of 6 cognitive abilities, but it also includes 5 more questions, three of them referring to the examination of attention and calculation, and the remaining two to language, with a maximum score of 35 points; this version maintains strong concordance with the original Mini Mental State Examination. As well as recording the overall MMCE score, the intersecting pentagon (IP) drawing component of the MMCE and Mini Mental State Examination and the writing sentence of the same tests were analysed separately. For the IP figure to be considered correct the patient must make a copy of the printed diagram. All 10 angles must be present and 2 must intersect; tremor and rotation are ignored. For the sentence writing, the researcher asks the patient to write a sentence, without dictating it. The sentence must contain a subject and a verb, and must make sense. Correct grammar and punctuation are not necessary.

**Table 1:** Multimorbidity functional criteria of the Andalusia Health Department.

**Category A**	
	1. Heart failure in functional NYHA class II or higher.
	2. Ischaemic heart disease.
**Category B**	
	1. Autoimmune diseases or systemic vasculitis.
	2. Chronic kidney disease defined by elevated creatinine (> 1.4 mg/dl in men or> 1.3 mg/dl in women) or proteinuria, maintained for 3 months.
**Category C**	
	1.-Chronic respiratory disease in clinically stable situation with dyspnoea measured with the mMRC scale ≥2 or FEV1 <65%, or SaO2 ≤ 90%.
**Category D**	
	1. Inflammatory bowel disease.
	2. Symptomatic liver disease (signs of portal hypertension or liver failure) or chronic activity.
**Category E**	
	1. Cerebrovascular attack.
	2. Motor neurological disease that causes a permanent deficit limitation for basic daily life activities. (a)
	3. Neurological disease with permanent cognitive impairment, at least minimally moderated (b).
**Category F**	
	1. Symptomatic peripheral artery disease.
	2. Diabetes mellitus with proliferative retinopathy or symptomatic neuropathy. (c)
**Category G**	
	1.-Chronic anaemia with digestive loss or acquired blood disorder with Hb <10 g/dl, in at least two determinations separated by three months.
	2. Solid or haematologic neoplasia not susceptible to active treatment with curative purposes.
**Category H**	
	1.-Chronic osteoarticular disease that causes a limitation of basic life activities.
	Excluded: patients in a transplant program, and those on dialysis or with AIDS.
	a) Barthel Index <60 points.
	b) Barthel Index <60 points and/or cognitive impairment minimally moderated (5 or more errors in Pfeiffer test).
	c) Presence of proliferative retinopathy, albuminuria, cerebral stroke, or symptomatic neuropathy.

Delirium was diagnosed with the confusion assessment method and dysphagia was evaluated by a speech therapist [21].

Measurement of the functional parameters, FEV_1_, FEV_6_, and FEV_1_/FEV_6_ ratio, was performed using the portable COPD-6 device (model 4000, Vitalograph Ltd., Ennis, Co. Clare, Ireland). The patient was required to perform manoeuvers in a similar way to those used in forced spirometry, with the difference that it was only necessary to maintain exhalation for the first 6 seconds. Measurements were repeated a maximum of 8 times, or until at least 3 reliable measurements were achieved [13]. The best values obtained for each patient were recorded. If 3 valid manoeuvers were not achieved after 8 attempts the patient was considered unable to perform the technique.

Subsequently, patients who were able to use the device correctly underwent conventional spirometry, using a portable spirometer (Datoespir micro, Sibelmed, Barcelona Spain). The test was performed in accordance with the Spanish Society of Pulmonology and Thoracic Surgery (SEPAR) guidelines [22]. Of the first 20 patients who were unable to perform FEV1/FEV6, none could perform the conventional spirometric manoeuvers correctly, so we did not attempt the test with the other patients unable to perform the COPD-6 procedures. If they were not able to maintain expiration for 6 seconds, it was considered that they could not do a complete forced expiration. The manoeuvers to determine FEV_1_ are similar for the two techniques. COPD-6 maneuvers were performed by two of the principal investigators (SK, PA), that are previously trained in COPD-6 use. Conventional spirometry was performed by a nurse specialized in pulmonary function tests. Respiratory maneuvers were performed without changing the treatment of the patients. Specifically, in patients under bronchodilator treatment, the values collected for COPD-6 and conventional spirometry refer to post-bronchodilator measures.

The exclusion criteria were death during hospitalization or presenting other reasons, apart from cognitive or functional impairment, which prevented the realization of FEV_1_/FEV_6_ (e.g., tracheotomy, facial paralysis....).

### Statistical analysis

Qualitative variables were expressed as absolute frequencies and percentages, while quantitative variables were summarized as mean and standard deviation (SD) in the case of normally distributed or median and interquartile range 25–75%. Comparison among means was made with the ANOVA test for independent samples with a parametric distribution or non-parametric test (Mann-Whitney U or Kruskal-Wallis) for variables not distributed normally. When the independent variable had more than 2 conditions, the Bonferroni or Games-Howell tests were used according to the homogeneity of the variances. Either the x2 test or the Fisher exact test was used for the comparison of proportions. Correlations between quantitative variables were determined with the Pearson or Spearman’s correlation test. Multivariable logistic analysis for the identification of independent correlates of outcomes was performed with stepwise linear regression models. Variables entered in the model were chosen based on univariant analysis results.

### Receiver operating characteristic (ROC) curves and their area under the curve

(AUC) were also analysed. The comparison of AUC was performed with the DeLong method. Statistical significance was determined for p-values below 0.05.

In the first approach using analysis of paired means, with a bilateral contrast, an usual nominal level of 0.05 (Type I error, alpha) and a power of 0.9 (Type II error, beta, of 0.1) the number of patients needed would be 199. Following the guides for reporting statistics in observational studies (STROBE), no multitesting corrections were applied. The analysis was performed using MedCalc Statistical Software for Windows (MedCalc Software bvba. Belgium)

The STROBE check-list for cross-sectional studies is detailed in a supporting information file “S2 Table”. The researchers read and gave written explanations of the study and informed consent to the patient, and in case of impossibility due to physical or cognitive impairment to the caregiver. The signature was always made in the presence of the researcher and the patient. Both documents, the written explanations and informed consent, were approved by the ethics committee.

The protocol was registered in ISRCTN with the number ISRCTN10703543. The study was classified by the Spanish agency of medicines and health products as an observational study and was approved by the Ethics and Clinical Trials Committee of University Hospital of the Mutua de Terrassa.

## Results

**Fig 1:**
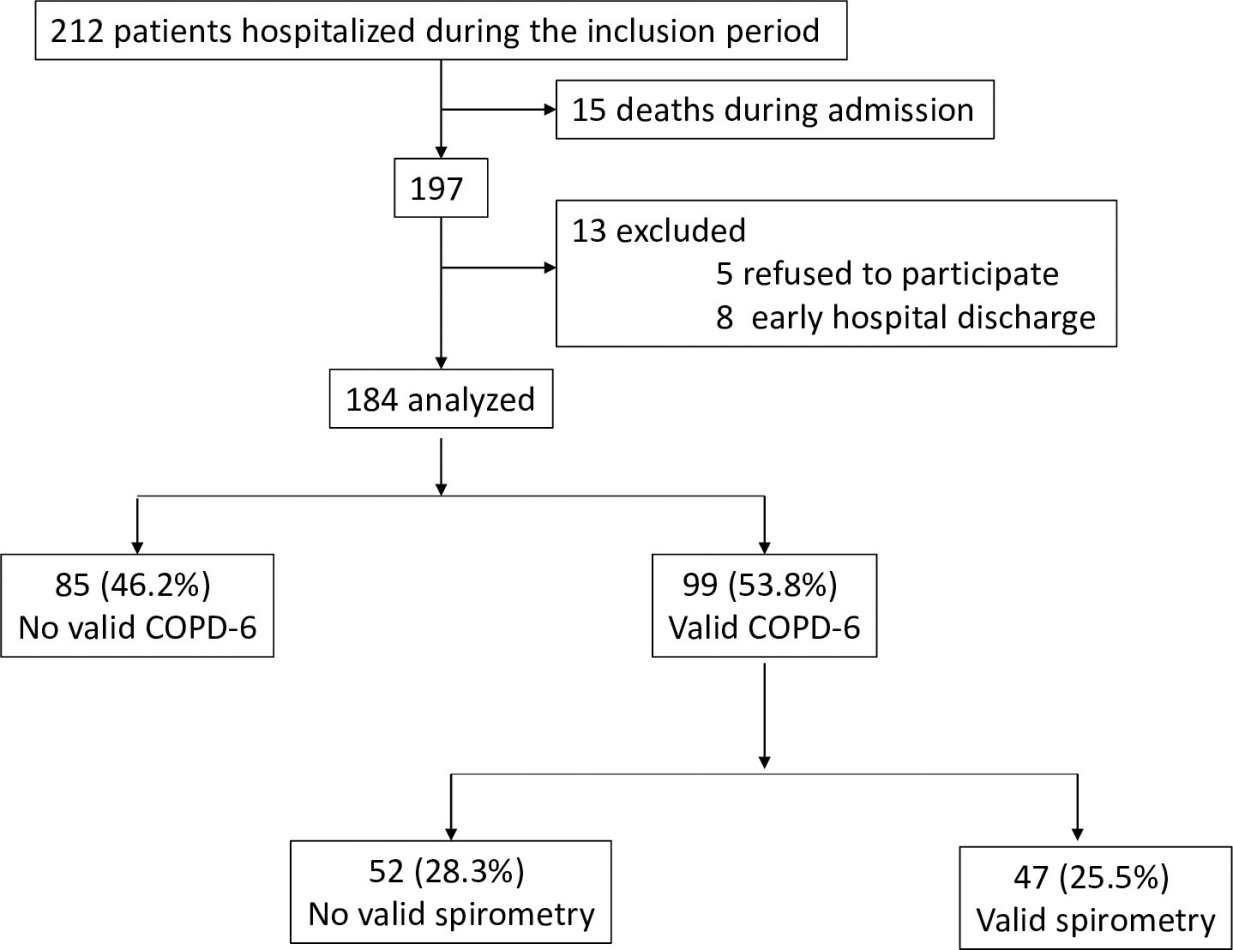
Flowchart of participants.

Overall, 197 patients met the inclusion criteria, of whom 184 were finally included. Thirteen patients (6.6%) were excluded due to missing data or not giving informed consent “Fig 1.” Excluded patients had better functional capacity and lower cognitive impairment according to the Barthel index and the Pfeiffer and MMCE scales, without differences in terms of age, gender, or Charlson index.

**Fig 2:**
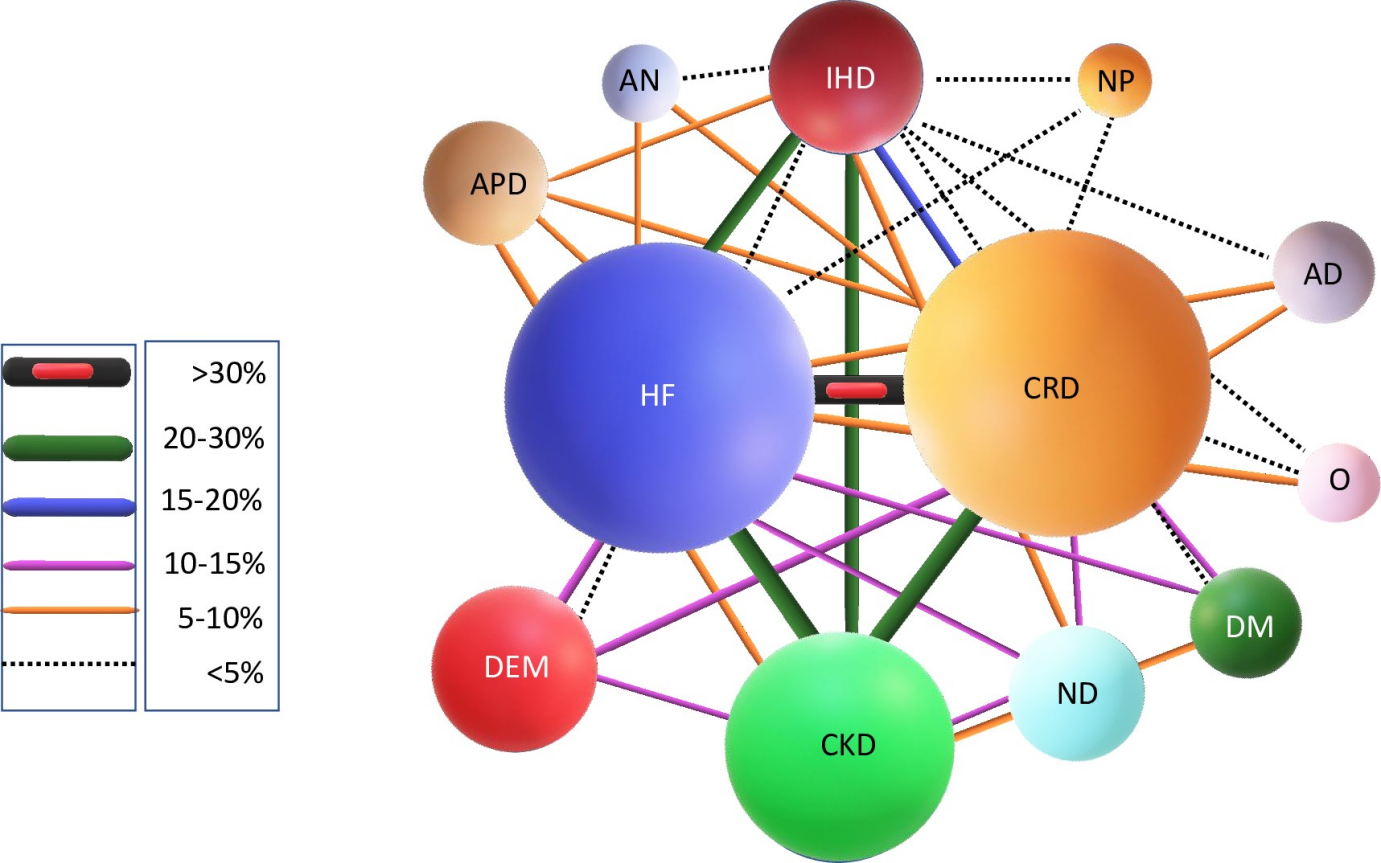
Correlation between different chronic diseases, according to multimorbidity scale. The size of the spheres represents the prevalence of diseases (HF = Heart failure, CRD = Chronic respiratory diseases, IHD = Ischaemic heart disease, CKD = Chronic kidney diseases, DEM = Dementia, ND = Neurological diseases, DM = Diabetes mellitus, PAD = Peripheral arterial disease, NP = Neoplastic diseases, AD = Autoimmune diseases, O = Osteoarticular diseases). Joined lines represent the strength of the correlation expressed as a percentage of the total.

Mean age of the studied population was 79.61 years (SD: 12.42; Interquartile range 25%-75%: 74–88), and 101 (55%) were males. The mean for chronic diseases in the multimorbidity scale was 3.1 (SD: 1.57), with the most frequent being heart failure (56.3%) and chronic respiratory diseases (54.33%). The mean of the Charlson index without age adjustment was 4.32 (SD: 2.38). Demographic and clinical characteristic are detailed in Table 2. The most frequent combination of chronic diseases was the coexistence of heart failure with chronic respiratory diseases (65 patients, 35%), heart failure with renal failure (49 patients 26.7%), chronic respiratory disease with renal failure (41 patients, 22.8) and heart failure with ischaemic heart disease (36 patients, 19.7%). The relationship between the various chronic diseases is shown graphically in “Fig 2”.

**Table 2:** Comparison of patients who can and cannot correctly complete the COPD-6 manoeuvers.

VARIABLE			p-value
	Non-valid COPD-6 (n = 85)	Valid COPD-6 (n = 99)	
Age (SD)	81.26 (13.20)	78.19 (11.58)	0.004
Gender			
Men	62 (62.6%)	39 (45.9%)	0.017
Women	37 (37.4%)	46 (54.1%)	
Barthel (SD)	36.48 (33.69)	70.39 (27.67)	<0.0001
Pfeiffer (SD)	5.98 (4.03)	1.62 (2.07)	<0.0001
MMSE (SD)	16.44(11.91)	28.15 (6.57)	<0.0001
MMCE sentence incorrect	71 (83.5%)	27 (27.3%)	<0.0001
MMCE pentagons incorrect	71 (83.5%)	41 (41.4%)	<0.0001
Length of admission, days (SD)	9.64 (5.99)	10.07 (6.13)	0.3
Domiciliary drugs (SD)	8.55 (4.23)	9.03 (4.05)	0.4
Multimorbidity criteria (SD)	3.09 (1.64)	3.09 (1.51)	0.9
Charlson age adjusted (SD)	7.98 (2.99)	7.59 (2.71)	0.3
Charlson not adjusted	4.42 (2.63)	4.23 (2.16)	0.5
PROFUND	14.86 (5.72)	7.92 (5.43)	<0.0001
Delirium	54 (63.5%)	25 (25.3%)	<0.0001
Dysphagia	52 (61.2%)	19 (19.4%)	<0.0001

A total of 85 patients (46.2%) were unable to perform valid manoeuvres with COPD-6 to measure FEV_1_/FEV_6_ ratio. The other 99 patients were able to correctly perform FEV_1_/FEV_6_ and of them 47 (47.5%) were also able to complete a spirometry. Therefore, 28.3% of the total number of patients included were able to perform FEV_1_/FEV_6_ with the handheld COPD-6 device but not with conventional spirometry “Fig 1”.

The researchers considered the main cause of being unable to correctly perform the FEV_1_/FEV_6_ to be dementia in 67.5% of the cases, functional impairment in 22.5%, and lack of collaboration in 10%.

**Fig 3:**
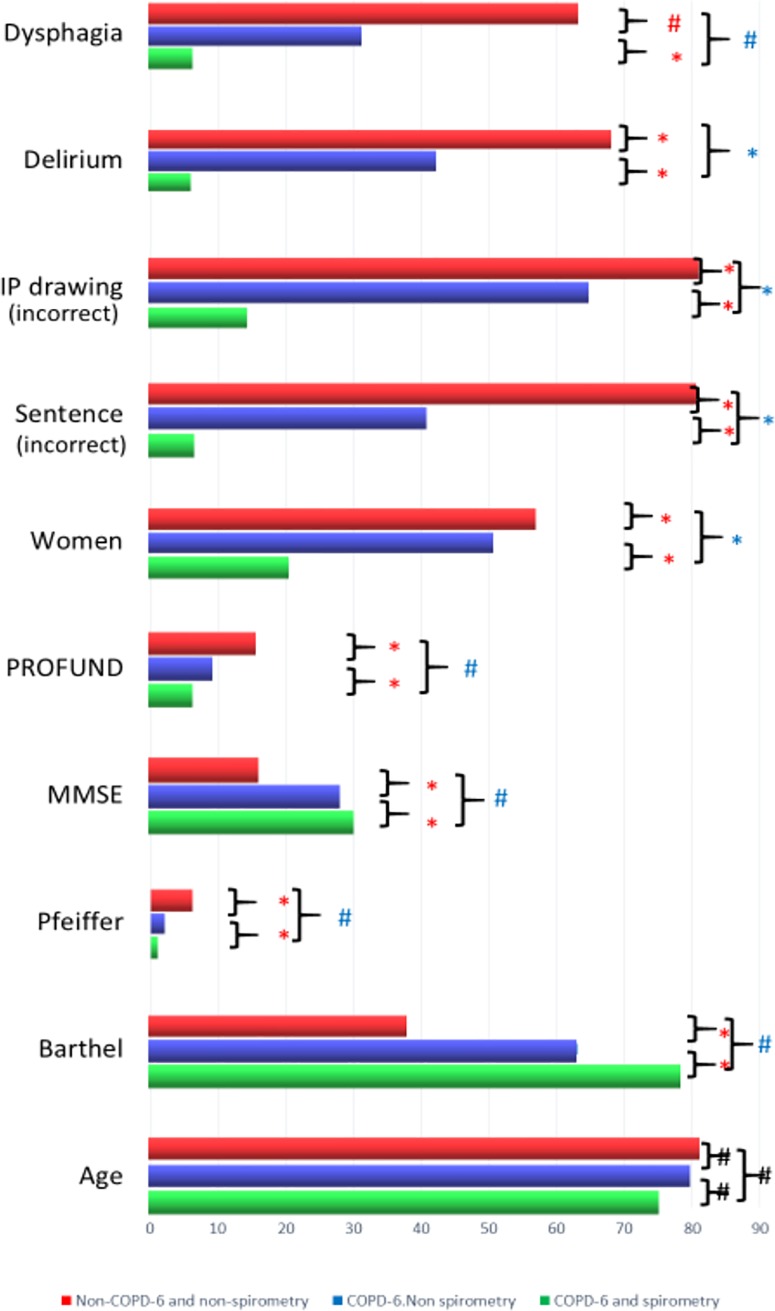
Differences between variables and ability to perform respiratory manoeuvers. *p<0.0001; # p<0.05. Sentence = unable to correctly complete sentence of MMCE. IP drawing = unable to correctly produce IP drawing of MMCE. * # red = bivariate analysis. * # blue = adjusted for multiple variables.

The differences between the 3 groups of patients (1. patients unable to perform valid COPD-6 manoeuvers; 2. patients with valid COPD-6 and incorrect conventional spirometric procedure, and 3. patients able to perform both techniques correctly) were significantly and linearly related with age, functional capacity measured with the Barthel index, cognitive impairment assessed with the Pfeiffer and the MMCE, and scores on the PROFUND prognostic scale (all p<0.05). Only 9% of patients with an MMEC of < 24 points could perform a valid spirometry. Of the 35 patients with an MMEC < 24 points unable to perform spirometry, 12 (34%) could complete FEV_1_/FEV_6_ manoeuvers. Women, those who could not correctly complete the sentence or PI figure on the MMCE, and those who presented delirium or dysphagia during admission were also less likely to successfully complete both COPD-6 and conventional spirometry (all p<0.0001). No differences were found in the Charlson index, multimorbidity scale, number of chronic domiciliary drugs, or length of stay between those patients able to and those not able to perform respiratory manoeuvers “Fig 3”.

**Fig 4:**
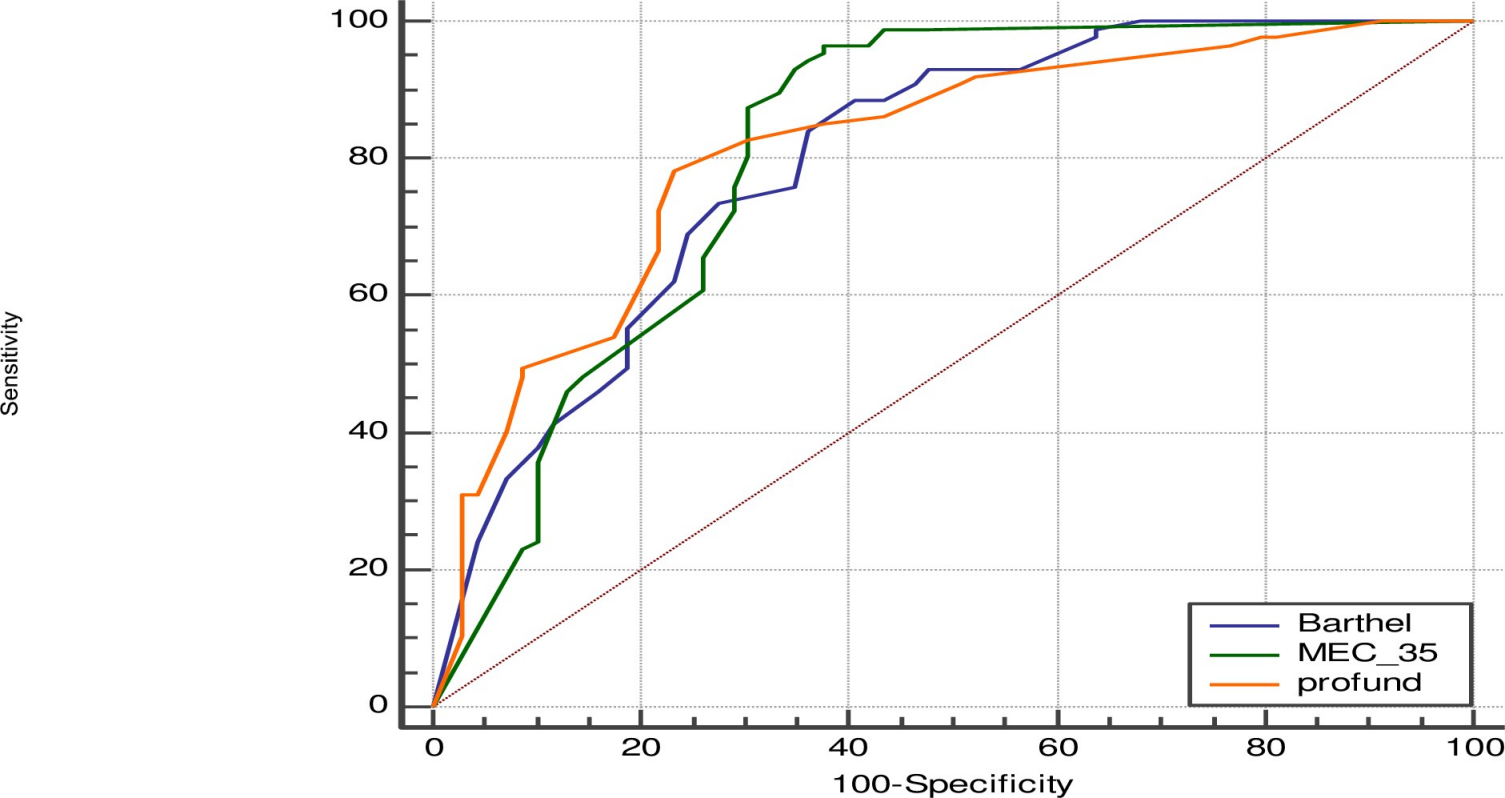
Comparison of ROC curves for Barthel (blue line), MEC (green line), and PROFUND (orange line), and probability of completing FEV_1_/FEV_6_ manoeuvers.

In a separate analysis, patients unable to perform the manoeuvers of COPD-6 acceptably compared with those who could determine the FEV_1_/FEV_6_ correctly were significantly older, with lower functional capacity, worse scores on scales of cognitive impairment, higher scores on the PROFUND index, and greater incidence of delirium and dysphagia during the hospital stay (Table 3). In this analysis the AUC for the capacity to correctly perform COPD-6 manoeuvers was 0.79 with a 95% confidence interval (CI) of 0.69–0.89 for the MMCE, 0.78 (CI 95%: 0.71–0.85) for the Barthel index, and 0.80 (CI 95%: 0.74–0.87) for the PROFUND scale “Fig 4”.

**Table 3:** Comparison of patients with valid COPD-6 who can and cannot correctly complete conventional spirometric test.

VARIABLE			p-value
	Non-valid spirometry (n = 52)	valid spirometry (n = 47)	
Age (SD)	81.01 (9.54)	75.02 (12.84)	0.009
Gender			
Men	25 (40.3%)	37 (59.7%)	0.001
Women	27 (73%)	10 (27%)	
Barthel (SD)	63.27 (24.41)	78.27 (29.14)	0.006
Pfeiffer (SD)	2.27 (2.19)	0.89 (1.67)	0.0001
MMSE (SD)	27.04 (6.34)	29.67 (6.67)	0.03
MMCE sentence incorrect	21 (77.8%)	6 (22.2%)	0.002
MMCE pentagons incorrect	34 (82.9%)	7 (17.1%)	<0.0001
Length of admission, days (SD)	11.33 (7.74)	8.58 (2.81)	0.4
Domiciliary drugs (SD)	8.32 (3.89)	9.88 (4.10)	0.65
Multimorbidity criteria (SD)	3.26 (1.65)	2.91 (1.32)	0.28
Charlson age adjusted (SD)	8 (2.55)	7.13 (2.84)	0.1
Charlson not adjusted	4.13 (2.16)	4.33 (2.18)	0.5
PROFUND	9.38 (5.75)	6.15 (4.48)	0.04
Delirium	22 (88%)	3 (12%)	<0.0001
Dysphagia	16 (84.2%)	3 (15.8%)	0.002
FEV_1_ (ml)	1.195 (648)	1.001 (614)	0.03
FEV_6_ (ml)	1.671 (784)	1.386 (717)	0.04
FEV_1_/FEV_6_	69.7 (15.3)	75.7 (12.5)	0.04

The respective ROC curves of these variables were similar without statistical significance. In the multivariate model, after adjustment for age, gender, Barthel, and PROFUND, only MMEC maintained statistical significance (p<0.0001). Patients unable to correctly complete the MEC sentence or IP figure had less probability of completing valid COPD-6 manoeuvers (both p<0.0001) with a sensitivity and specificity of 72.7% and 84.7% for the sentence and a sensitivity of 63.7% and specificity of 81.7% for the IP figure, respectively.

The same variables were associated with the inability to complete a valid spirometry test in those patients who previously could correctly perform COPD-6 determinations (Table 3). After adjustment for MMCE, Barthel and PROFUND index, only age retained statistical significance (p = 0.01). In this analysis patients who were able to perform both techniques compared with those who could only complete COPD-6 had better FEV_1_ and FEV_6_ but a higher degree of airflow obstruction measured with the FEV_1_/FEV_6_ ratio (Table 3).

**Fig 5:**
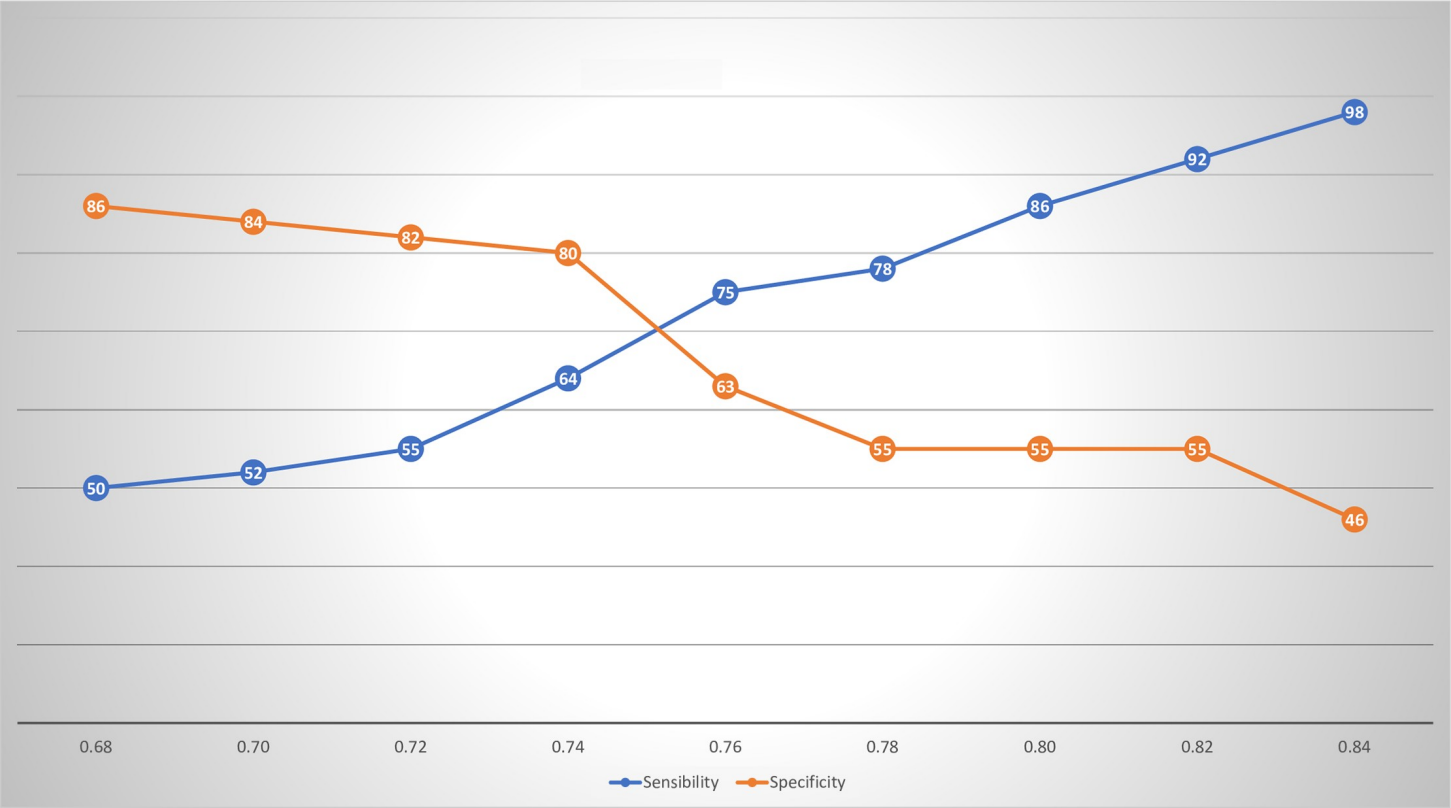
Sensitivity and specificity of different thresholds of FEV_1_/FEV_6_ and airway obstruction defined as FEV_1_/FVC <0.7.

The agreement between the values for FEV_1_ measured during hospitalization with COPD-6 and those observed in the conventional spirometry was good (Pearson correlation: r: 0.71; p<0.0001). Of the 52 patients with a valid COPD-6 who could not perform the spirometry, in 29 cases a previous spirometry could be recovered, so in 76 cases the diagnosis of obstruction defined by FEV_1_/FVC <0.7 could be compared with the values obtained in FEV_1_/FEV_6_. The correlation between FEV_1_/FEV_6_ and FEV_1_/FVC was confirmed as good (r:0.643; p<0.0001). The different values for sensitivity and specificity for the diagnosis of airflow obstruction defined as FEV_1_/FVC <0.7 and several cut-offs of the FEV_1_/FEV_6_ between 0.68 and 0.84 are shown graphically in “Fig 5”.

Finally, using the cut-off point recommended in the literature for the diagnosis of airway obstruction with FEV_1_/FEV_6_ (<0.73), 43 (43.4%) patients met the obstruction criteria, of whom 15 (34%) were not previously known.

## Discussion

The data from the present study corroborate the difficulty of correctly performing the expiratory manoeuvers necessary to diagnose airflow obstruction during hospital admission in a population that is increasingly prevalent: elderly patients with multimorbidity. This incapacity is closely related with the presence of cognitive and functional impairment, especially frequent in these patients, but not with the number of multimorbidity criteria. Our study also reinforces the usefulness of FEV_1_/FEV_6_ in a large number of patients unable to perform conventional spirometry and confirms the high rate of underdiagnosis of pulmonary obstruction in these patients.

The present study, together with a small previous pilot study conducted by our group, is the first to assess the difficulty in performing spirometry and also the usefulness of FEV_1_/FEV_6_ in hospitalized patients with multimorbidity [12]. The most frequent definition of multimorbidity is the coexistence in the same patient of two or more chronic diseases [4,23]. Another approach recommended by the NICE guidelines is to consider, in addition to the number of chronic diseases, other factors such as fragility, polypharmacy, and complexity of care [24]. In our case the definition of multimorbidity was based on a scale that accounts not only for the number of chronic diseases but also their severity and impact on patients' functional capacity [14]. Additionally, we used one of the most recognized comorbidity scales, the Charlson index [16]. Our data show that multimorbidity by itself is not associated with a higher prevalence of patients unable to perform spirometric manoeuvers. The best predictors for the inability to perform a quality spirometry are age, cognitive impairment, and physical functional disability, all of which are also closely related to each other.

With respect to age, the percentage of elderly subjects able to perform a classical spirometry with the established quality criteria varies widely depending on the population studied, ranging between 22% and 93% [9,10,25–31]. Some of these studies selected populations of healthy elderly and show that age by itself does not prevent the performance of quality spirometry, although obviously the prevalence of cognitive impairment, chronic diseases, and frailty rises with age. In light of this, Pezzoli et al., in a study conducted in ambulatory elderly patients recruited in a geriatric outpatient unit, found that 80% were able to perform a quality spirometry, although the time needed to do so was 20–30 minutes—practically double the time needed for young patients. In this study the mean age of the patients was 75 and the prevalence of cognitive impairment (defined as an MMSE <21) was as low as 1.5% [25]. Similar results were reported by Bellia et al. in a cohort of 1,622 outpatients over 65 years of age recruited in geriatric institutions [9]. Our results are more comparable to those of Allen et al., in a cohort of patients hospitalized in a rehabilitation ward with a mean age of 80. In that study the authors showed that only 22% of patients were able to complete a spirometry with the validity criteria established by the American Thoracic Society, although 67% were able to perform the FEV_1_ manoeuvers correctly. Of note, this prevalence should be even lower since patients with an MMSE <11 were excluded from the study [10]. The exclusion of patients with different degrees of dementia—a characteristic common to all previously published studies—generates a selection bias and makes it difficult to uncover the true percentage of patients capable of performing the technique. In our study, we decided not to exclude patients with dementia in order to learn the real percentage of patients unable to perform spirometry, although obviously those with greater cognitive impairment were already expected to be unable to collaborate in the realization of the technique. Of note, in our study age was non-significant after multivariate analysis for the possibility of performing COPD-6, but retained its significance after adjustment for Barthel, MMCE, and PROFUND for the capacity to successfully complete a spirometry in patients who previously were able to complete manoeuvers for FEV_1_/FEV_6_.

The relationship between cognitive impairment and the inability to perform spirometry manoeuvers is well known. In fact, some authors have suggested that dementia screening should be performed in patients who, for no obvious reason, cannot perform a valid spirometry test [29]. However, the confirmation of an obstructive pattern is necessary to confirm some respiratory diseases such as COPD. This leads to a paradoxical situation: while longitudinal studies show an increase in the incidence of dementia in patients with COPD, in cross-sectional studies the prevalence of dementia is relatively small, given that the patients cannot perform quality spirometry and are therefore excluded from the studies [15,32].

The test most widely used in previous research to measure the relation between cognitive impairment and the impossibility of performing a complete spirometry is the MMSE or its adaptations [26–31]. In this test, values lower than 24/30 are usually considered indicative of cognitive impairment—a threshold shared with the Spanish version. Our study confirms these previous data in that only 9% of patients with an MEC of < 24 points could perform a valid spirometry. Our study also reinforces the results of two previous studies of the usefulness of the sentence and the IP drawing of MMSE as rapid detection tests for the impossibility of performing spirometry [10,27]. Only 6% of patients with an incorrect sentence and 7% with an incorrect IP drawing were able to complete the technique.

The main impediment to performing conventional spirometry in these patients is the inability to maintain expiratory flows until completion of the forced expiration. Handheld spirometer devices allow assessment of the airflow obstruction based only on the first 6 seconds of expiration, so the quotient is obtained by dividing the FEV_1_/FEV_6_ instead of the classically used FEV1/FVC. Their usefulness in screening for COPD and concordance with the values obtained in classical spirometry have been well demonstrated in several studies conducted in the general population [13, 33–38]. In fact, FEV_1_/FEV_6_ is a more reliable index than FEV_1_/FVC in longitudinal studies, because FVC is more variable for the differences in the duration of the forced exhalation [39]. In elderly patients the prognostic value of a low FEV_1_/FEV_6_ is comparable to that of a low FEV_1_/FVC and FEV_1_ [40].

Beyond its usefulness in obstruction screening, another less explored advantage of FEV_1_/FEV_6_ is the capacity to recover a percentage of patients unable to perform a complete spirometry, although information on this point is scarce. In a study performed in elderly patients, FEV_1_/FEV_3_ allowed recovery of 25% of patients unable to perform spirometry, including some with an MMSE <24 [10]. In another study performed in 1,531 outpatients with a mean age of 74, valid FVC measurements were achieved in 56.9% with the spirometric test, while this percentage increased to 82.9% with the FEV_6_ [11]. In yet another study performed on hospitalized patients with multimorbidity, 65% of the patients successfully performed FEV_1_/FEV_6_ with another handheld device (Piko 6 nSpire Health, Inc, Germany) [12]. In our study of the 99 patients with a valid FEV_1_/FEV_6_, 47 (47.5%) were unable to perform complete expiratory manoeuvers, and 34% of the patients with an MMEC < 24 points who were unable to perform spirometry were able to complete FEV_1_/FEV_6_ manoeuvers. Similarly, the percentage of patients with an incorrect IP drawing increased from 22% for the determination of FEV_1_/FEVC to 78% for FEV_1_/FEV_6_.

In our study, we compared the values for FEV_1_ measured consecutively with COPD and spirometry only in those patients who were able to perform both techniques during the hospital stay in question, since FEV_1_ values vary with time and exacerbations. Nevertheless, to confirm the classic diagnosis of airway obstruction (FEV_1_/FVC <0.7), we used the current spirometry data in patients able to perform a valid spirometry during the stay in question or, alternatively, considered a previous spirometry from the clinical history if one was recovered. In both cases, our study confirms the strong correlation between values observed in the FEV_1_ measured with the two methods and the diagnosis of airflow obstruction measured with the FEV_1_/FEV_6_ and FEV_1_/FVC.

Other predictors of the inability to perform short, complete spirometric expiration manoeuvers in our study were female gender and the presence of delirium or dysphagia during admission. The increased difficulty of elderly women in completing expiratory manoeuvers has already been reported in other studies [11]. As to delirium and dysphagia, they are markers of frailty and are frequent during hospital stays in elderly patients with chronic diseases.

Our study has some limitations that need to be considered. First, it was performed in a single hospital with a specific ward for attention to patients with multimorbidity. Therefore our results may not be extrapolated to patients hospitalized in other services. However, the percentage of patients capable of performing spirometry is comparable to other studies performed in a similar population [10]. Second, our research was carried out during a hospital admission—a situation that generates an increase in weakness with impaired functional capacity and a higher incidence of delirium and dysphagia [41–44]. It is likely that an indeterminate number of patients who could not complete the spirometry manoeuvers during a hospital stay could do so in the following months if their functional capacity improved. Although the clinical guidelines recommend performing spirometry in a stable phase, at least one month after hospitalization, in this population this is often not feasible, due to difficult logistics, hospital readmissions, and death. In the follow-up of the present cohort, 30% of the patients were deceased or had been readmitted during the month after hospital discharge. This approach allows us to avoid the unresponsive bias. However, the diagnosis of obstruction performed during admission with the COPD-6 is closely related to the values obtained with conventional spirometry at 6 weeks after discharge. In two recent studies performed in hospitalized patients with FEV_1_/FVC < 70% during admission the obstructive airflow pattern in conventional spirometry persisted during the follow-up. Of note, in these studies nearly half of the subjects did not attend the follow-up visit [45,46]. After finding that the first 20 patients unable to perform COPD-6 were also unable to perform spirometric manoeuvers, we did not attempt to perform spirometry in the rest of the patients without a valid COPD-6 determination. This is reasonable, since if they were not able to maintain expiration for 6 seconds it was considered that they could not achieve a complete forced expiration.

Finally, in our study, we did not analyse the bronchodilator test for two reasons. First so as not to fatigue patients after the maneuvers of COPD-6 and spirometry. Performing the bronchodilator test would double the number of procedures. Second, it is advisable to stop bronchodilator treatment from performing the bronchodilator test in patients who are not stabilized. However, practically all patients classified as obstructive performed both measurements (COPD-6 and conventional spirometry) under bronchodilator treatment.

In conclusion, our study demonstrates the difficulty of performing spirometric manoeuvers in a significant percentage of elderly patients with multimorbidity hospitalized for decompensation of their chronic diseases. Nevertheless, a significant number of these patients can perform simpler manoeuvers that also correlate well with conventional spirometry data, such as FEV_1_/FEV_6_.

## Supporting information

S1 Table PROFUND index. (DOCX)

S2 Table STROBE statement. (DOCX)

S3 Table Comparison of patient characteristics according to their ability to perform respiratory manoeuvers. (DOCX)
