# Supplementary material for: Utility of FEV1/FEV6 index in patients with multimorbidity hospitalized for decompensation of chronic diseases
Source: PLoS One. 2019 Aug 2;14(8):e0220491. doi: 10.1371/journal.pone.0220491 (PMC6677320; doi:10.1371/journal.pone.0220491)
Supplement: S1 Table — (DOCX) [file pone.0220491.s001.docx]

S1 Table

| PROFUND SCORE | points |
| --- | --- |
| >84 years | 3 |
| Active neoplasm | 6 |
| Dementia (Pfeiffer >5) | 6 |
| III-IV NYHA | 3 |
| 3-3 mMRC | 3 |
| Delirium during admission | 3 |
| Hb<10 | 3 |
| Barthel <60 | 4 |
| No caregiver or caregiver other than partner | 2 |
| 4 hospitalizations in the previous year | 3 |
| TOTAL |  |
