# Supplementary material for: Utility of FEV1/FEV6 index in patients with multimorbidity hospitalized for decompensation of chronic diseases
Source: PLoS One. 2019 Aug 2;14(8):e0220491. doi: 10.1371/journal.pone.0220491 (PMC6677320; doi:10.1371/journal.pone.0220491)
Supplement: S3 Table — (DOCX) [file pone.0220491.s003.docx]

S3 Table

Comparison of patient characteristics according to their ability to perform respiratory manoeuvers.

| VARIABLE |  |  |  | p-value |
| --- | --- | --- | --- | --- |
|  | Non-COPD-6  N=85 | COPD-6 yes  and non-valid spirometry  n=52 | COPD-6 yes  and valid spirometry  n=47 |  |
| Age (SD) | 81.25 (13.20) | 81.06 (9.54) | 75.02 (12.84) | 0.05 |
| Gender |  |  |  |  |
| Women | 46 (54.1%) | 27 (51.9%) | 10 (21.3%) | 0.001 |
| Barthel (SD) | 36.48 (33.68) | 63.26 (24.41) | 78.27 (29.13) | <0.05 |
| Pfeiffer (SD) | 5.97 (4.03) | 2.26 (2.19) | 0.89 (1.67) | <0.05 |
| MMCE (SD) | 16.44 (11.91) | 27.04 (6.34) | 29.66 (6.66) | <0.05 |
| MMCE sentence incorrect | 71 (83.5%) | 21 (40.4%) | 6 (6.1%) | <0.0001 |
| MMCE pentagons incorrect | 71 (83.5%) | 34 (65.4%) | 7 (14.9%) | <0.0001 |
| Length of admission, days (SD) | 9.64 (5.92) | 11.33 (7.74) | 8.58 (2.81) | n.s. |
| Domiciliary drugs (SD) | 8.55 (4.23) | 8.3 (3.89) | 9.88 (4.11) | n.s |
| Multimorbidity criteria (SD) | 3.09 (1.64) | 3.25 (1.64) | 2.91 (1.32) | n.s. |
| Charlson age adjusted (SD) | 7.98 (2.99) | 8.00 (2.55) | 7.13 (2.84) | n.s |
| Charlson not adjusted | 4.42 (2.62) | 4.32 (2.17) | 4.13 (2.16) | n.s |
| PROFUND | 14.85 (5.72) | 9.38 (5.74) | 6.14 (4.48) | 0.05 |
| Delirium | 54 (63.5%) | 22 (42.3%) | 3 (6.4%) | <0.0001 |
| Dysphagia | 52 (61.2%) | 16 (30.7%) | 3 (6.4%) | <0.0001 |

SD= Standard deviation; n.s. = non-significant.
